# Concentration-Dependency and Time Profile of Insulin Secretion: Dynamic Perifusion Studies With Human and Murine Islets

**DOI:** 10.3389/fendo.2019.00680

**Published:** 2019-10-02

**Authors:** Oscar Alcazar, Peter Buchwald

**Affiliations:** ^1^Diabetes Research Institute, Miller School of Medicine, University of Miami, Miami, FL, United States; ^2^Department of Molecular and Cellular Pharmacology, Miller School of Medicine, University of Miami, Miami, FL, United States

**Keywords:** beta cell function, concentration-response, glucose-stimulated insulin secretion, islet assessment, perifusion, stimulation index, type 1 diabetes

## Abstract

The detailed characterization and quantification of the kinetics of glucose-stimulated insulin secretion (GSIS) by normal pancreatic islets is of considerable interest for characterizing β-cell dysfunction, assessing the quality of isolated islets, and improving the design of artificial pancreas devices. Here, we performed dynamic evaluation of GSIS by human and mouse islets at high temporal resolution (every minute) in response to different glucose steps using an automated multichannel perifusion instrument. In both species, insulin responses were biphasic (a transient first-phase peak followed by a sustained second-phase), and the amount of insulin released showed a sigmoid-type dependence on glucose concentration. However, compared to murine islets, human islets have (1) a less pronounced first-phase response, (2) a flat secretion rate during second-phase response, (3) a left-shifted concentration response (reaching half-maximal response at 7.9 ± 0.4 vs. 13.7 ± 0.6 mM), and (4) an ~3-fold lower maximal secretion rate (8.3 ± 2.3 vs. 23.9 ± 5.1 pg/min/islet at 30 mM glucose). These results can be used to establish a more informative protocol for the calculation of the stimulation index, which is widely used for islet assessment in both research and clinical applications, but without an accepted standard or clear evidence as to what low- to high-glucose steps can provide better characterization of islet function. Data obtained here suggest that human islet functionality might be best characterized with a dynamic stimulation index obtained with a glucose step from a low of 4–5 to a high of 14–17 mM (e.g., G4 → G16).

## Introduction

In normal healthy subjects, blood glucose levels are maintained in a relatively narrow range between 3.0 and 9.0 mM (54–162 mg/dL), an observation now well-documented by continuous glucose monitoring (CGM) systems (1, 2). This is mainly achieved via a finely tuned control system that relies on insulin producing β-cells located in pancreatic islets to adjust their insulin secretion depending on the blood glucose levels. Glucose levels that define normoglycemia in a given species (glycemic set point) are species-specific as target glycemic levels vary among species; they are around 5 mM (90 mg/dL) in humans (3). While the biological determinants of the glycemic set point are still unclear and might involve multiple mechanisms, pancreatic islets seem to be able to act as a main glucostat and impose their glycemic set points (4). In response to a stepwise increase in glucose, insulin is released in a biphasic manner with a transient first-phase peak of 5–10 min followed by a more sustained second phase. Abnormalities in β-cell function are critical not only in type 1 (T1D), but also type 2 diabetes (T2D); hence, the accurate quantitative characterization of the kinetics of glucose-stimulated insulin secretion (GSIS) is of obvious interest for both T1D and T2D. It could help to better understand the process, assess β-cell function, and hence quantify progress toward disease onset in prospective patients. It is also of critical importance for the development of improved artificial (e.g., closed loop) and bioartificial pancreas systems (e.g., encapsulated islets).

Perifusion studies have been introduced in the late 1960s (5–7), and improved equipment and analytical techniques now allow the quantitative assessment of insulin release kinetics with adjustable temporal resolution under fully controllable concentrations of glucose, oxygen, and other secretagogues of interest. Since they allow the dynamic measurement of GSIS, they represent the most complex *in vitro* assay to assess the quality and function of isolated pancreatic islets and provide considerably more information-rich description than obtainable from static GSIS and corresponding stimulation indices (SIs). Dynamic perifusion is now routinely used to assess the quality and function of islets isolated for transplant or experimental purposes (8, 9); however, various non-standardized systems and protocols are being used including glucose steps involving diverse pairs of basal (low) and stimulating (high) concentrations. The aim of the present study was to exploit developments in perifusion equipment and insulin detection to quantify the dependence of insulin secretion on the incoming glucose step more accurately and use this to establish conditions that could best assess function for both human and murine islets. Stimulation indices (SIs, calculated as the ratio between the insulin secreted at high vs. low glucose) are widely used for islet assessment in both research and clinical applications, but there is no commonly accepted standard protocol or even clear evidence as to what glucose step should be used to obtain the best characterization of functionality. Studies here were performed with a fully automated machine with software-controlled customizable input for multiple parallel channels (4 × 3) that allows collection with adjustable temporal resolution.

## Materials and Methods

### Human Islets

Human pancreatic islet samples were procured from the Integrated Islet Distribution Program (IIDP) at City of Hope or from isolations performed at the Human Islet Cell Processing Facility at the Diabetes Research Institute (University of Miami, Miller School of Medicine, Miami, FL, USA). The islet isolation protocol, as part of the Clinical Pancreatic Islet Transplantation Study, was approved by the Institutional Review Board (IRB) of the University of Miami and the FDA. Human pancreases were isolated from deceased multi-organ donors for whom consent for transplantation was obtained by accredited organ procurement organizations (OPO) from the donor's families or next of kin. All samples tested here were from non-diabetic donors; characteristics of the human islet donors for the present study are summarized using standard checklists recommended for reporting human islet preparations used in research in Tables S2, S3. Mouse islets used were obtained and processed as described before (10, 11).

### Animal Housing and Islet Procedures

All animal studies were reviewed and approved by the University of Miami Institutional Animal Care and Use Committee. All procedures were conducted according to the guidelines of the Committee on Care and Use of Laboratory Animals, Institute of Laboratory Animal Resources (National Research Council, Washington DC, USA). Animals were housed in microisolated cages in Virus Antibody Free rooms with free access to autoclaved food and water at the Department of Veterinary Resources of the University of Miami. Islets were obtained from donor mice (10–12 week old, both male and female C57BL6/J, Jackson Lab, Bar Harbor, ME, USA) via mechanically enhanced enzymatic digestion followed by density gradient purification as previously described (10, 11). Briefly, animals were sacrificed under general anesthesia, and the pancreas was exposed and injected with Hanks' balanced salt solution (HBSS; Mediatech, Herndon, VA, USA) containing either 0.8 mg/mL collagenase type V (Sigma-Aldrich, St. Louis, MO, USA) or a mix of 0.2 mg/mL Liberase TL and 0.1 mg/mL DNase (Roche, Indianapolis, IN, USA) via the main bile duct until distension was achieved. Digestion was performed at 37°C for 10–15 min with gentle shaking and terminated by the addition of cold RPMI-10% fetal bovine serum (FBS) with 20 mM Hepes buffer, 1% penicillin-streptomycin, and 1% L-glutamine (all from Sigma-Aldrich). Mechanical disruption of the pancreas was achieved by passages through needles of decreasing gauge until release of islets was observed under a microscope; the tissue was filtered through a 450 μm mesh, and islets were purified on Euro-Ficoll (Mediatech) gradients by centrifugation at 400 g for 15–20 min, routinely yielding preparations of 90% purity.

Islet purity was assessed by dithizone (Sigma-Aldrich) staining, and islets were counted and scored using a standard algorithm for the calculation of 150 μm diameter islet equivalent (IEQ) number (12). Murine islets were cultured in complete CMRL 1066-based medium, which is CMRL 1066 (Mediatech; contains 1 g/L = 5.56 mM glucose) with 10% fetal bovine serum (FBS), 20 mM HEPES buffer, 1% penicillin-streptomycin, and 2 mM L-glutamine added (all from Sigma-Aldrich) at 37°C in 5% CO_2_ humidified incubator for 24–48 h prior to perifusions. Human islets were cultured in CMRL-1066 supplemented medium (Mediatech; contains 5.56 mM glucose, 25 mM HEPES, 1 g/L = 4.6 mM L-alanine–L-glutamine) with 2% human serum albumin (HSA) added, at 37°C in 5% CO_2_ humidified incubator for 24–48 h prior to perifusions.

### Islet Perifusions

The perifusion experiments (dynamic GSIS) were performed using a PERI4-02 machine (Biorep Technologies, Miami, FL, USA) that allows parallel perifusion for up to four independent channels as described before (11). For each experiment, 70 (mouse) to 100 (human) IEQ (all from the same islet isolation batch) were handpicked and loaded in Perspex microcolumns between two layers of acrylamide-based microbead slurry (Bio-Gel P-4, Bio-Rad Laboratories, Hercules, CA, USA) by the same experienced operator. Perifusing buffer containing 125 mM NaCl, 5.9 mM KCl, 1.28 mM CaCl_2_, 1.2 mM MgCl_2_, 25 mM HEPES, and 0.1% bovine serum albumin at 37°C with selected glucose or KCl (25 mM) concentrations was circulated through the columns at a rate of 100 μL/min. After 60 min of washing with low glucose (G3) solution for stabilization, islets were stimulated with the following sequence: 8 min of low glucose, 20 min of high glucose, 15 min of low glucose, 10 min of KCl, and 10 min of low glucose. Samples (100 μL) were collected every minute from the outflow tubing of the columns in an automatic fraction collector designed for a multi-well plate format. Islets and the perifusion solutions were kept at 37°C in a built-in temperature-controlled chamber while the perifusate in the collecting plate was kept at <4°C to preserve the integrity of the analytes. Insulin concentrations were determined with commercially available human and mouse ELISA kits, respectively (Mercodia Inc., Winston Salem, NC, USA). Values obtained with the human kit are in mU/L and were converted to μg/L using 1 μg/L = 23 mU/L per the manufacturer guidelines. Because accurately assessing islet mass (IEQ) is difficult (12, 13), to account for possible differences among islets in different channels, values were adjusted by up to 30% based on the response to KCl as described before (10, 11) using the area under the curve (AUC) in each column for normalization. All responses are scaled to 100 IEQ. Since only four different conditions could be tested in parallel, two consecutive perifusion runs were used for each islet batch to obtain all six conditions, with one condition (11 mM glucose) used as reference in both. This was used to ensure that the two consecutive runs are sufficiently similar; no further modification have been done to these data.

### Statistical Analyses

Data used here are averages of at least three samples for each condition. Curve fittings by non-linear regression were performed using GraphPad Prism (GraphPad, La Jolla, CA, USA). Dynamic stimulation indices (dSI) were calculated as the ratio between average insulin secretions during high- and low-glucose stimulation. Average stimulated insulin secretion was calculated for the entire 20 min of high glucose (minutes 13–32 to account for the delay), phase one for the first 5 min, and phase two for the last 13 min of high glucose.

## Results

### Mouse vs. Human Islets

**Figure 1:**
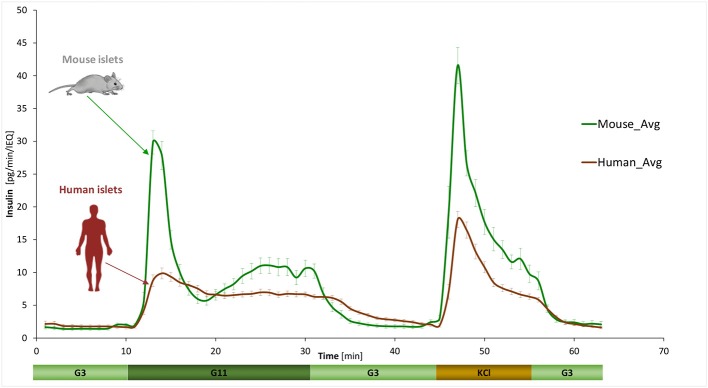
Dynamic GSIS in isolated human and murine islets. Average of all experimental data collected for free murine and human islets perifused using a low (3 mM; G3, 8 min) → high (11 mM; G11, 20 min) → low (3 mM; G3, 15 min) incoming glucose stimulation (plus 10 min KCl followed by G3) as shown. Automated PERI4-02 multichannel perifusion apparatus used (samples collected every minute; 0.1 mL/min flow rate, ~100 IEQ per channel). Data are average ± SEM from multiple isolations (*n* = 25 and 34 total samples for murine and human, respectively).

**Figure 2:**
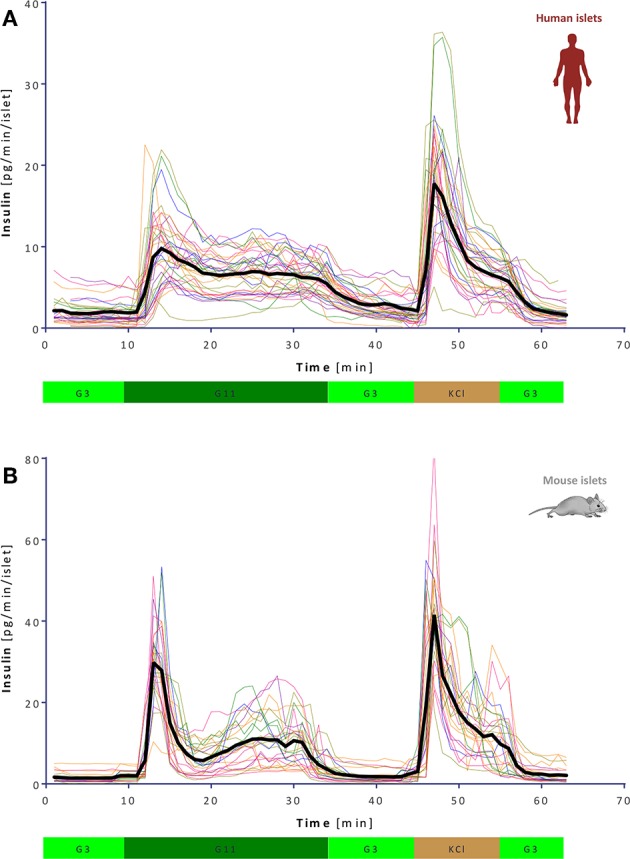
Variability of dynamic GSIS in isolated human and mouse islets. All experimental data for human **(A)** and mouse **(B)** islets perifused as before (G3 → G11 → G3 → KCl, see Figure 1) shown together with their overall average (thick black line; *n* = 34 and 25, respectively).

GSIS was assessed using a fully automated programmable multichannel perifusion apparatus (Figure S1) that allowed direct parallel comparison of the responses. A sudden stepwise increase in glucose caused typical biphasic responses (11, 14–18) in both murine and human islets with a transient first phase peak of 5–10 min followed by a more sustained second phase. However, there were clear differences. A first illustration is provided by the average curves shown in Figure 1 obtained from a large number of perifusions (*n* = 25 and 34) following our standard protocol (G3 → G11 → G3). While there was considerable variability among individual samples (Figure 2), overall averages are very consistent. They confirm our previous observation from a much smaller sample (11) that in response to a G3 → G11 glucose step, human islets secrete less insulin per islet mass (islet equivalent, IEQ) than murine islets and with a less pronounced first phase peak followed by a different second-phase plateau. Data (Figure 1) indicate about 3-fold differences during the first-phase peak (~10 vs. ~30 pg/IEQ/min) and 2-fold differences during the second-phase plateau (6.6 vs. 10.4 pg/IEQ/min). There is also an about 2-fold difference in the insulin release induced by KCl depolarization.

Furthermore, second-phase behaviors are also different: whereas, they are essentially flat in humans, there is a rising tendency in mouse islets. As verification, we performed perifusions with a more prolonged high-glucose phase (60 vs. 20 min) at two concentrations (G11 and G16.7). Results indeed confirmed that while the second-phase release remains constant in human islets, it increases over time in murine islets, possibly reaching saturation after some time (>0.5 h) especially at higher glucose concentrations (Figure S2). There is also a noticeable tendency toward an oscillatory pattern in insulin release, especially with murine islets, where the oscillations were more pronounced (had larger amplitudes) and had a periodicity of around 3 min.

### Concentration-Dependence of Dynamic GSIS

**Figure 3:**
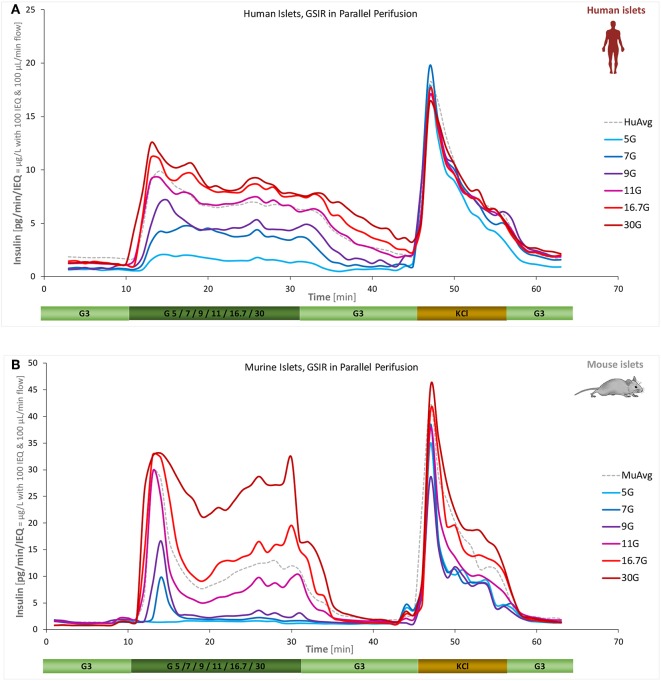
Concentration dependence of insulin secretion in murine and human islets. Summary of experimental data for free human **(A)** and murine islets **(B)** perifused using standard equipment and parallel stepwise incoming glucose stimulations (3 mM → 5/7/9/11/16.7/30 mM → 3 mM as indicated; plus 10 min KCl and 10 min low; corrected to ~100 IEQ per chamber; *n* = 3–12 per group). Averages of all data obtained in our labs for the G3 → G11 protocol are included as dashed gray lines for reference.

A main goal of the present work was to quantify the concentration-response of insulin secretion accurately, i.e., the effect of different glucose-steps on the magnitude and time-course of (biphasic) insulin secretion. This was investigated for both human and mouse islets using six different high-glucose steps (5, 7, 9, 11, 16.7, and 30 mM) starting from a low glucose baseline of 3 mM. Islets from the same batch were all perifused in parallel allowing a direct comparison of the differences due to the incoming glucose challenge. As Figure 3 shows, the essential biphasic character is maintained under almost all conditions, but there are clear differences between human and mouse secretion profiles. Human islets reach their maximal response at lower glucose: response at 16.7 mM was already saturated; by no means the case for mouse islets.

**Figure 4:**
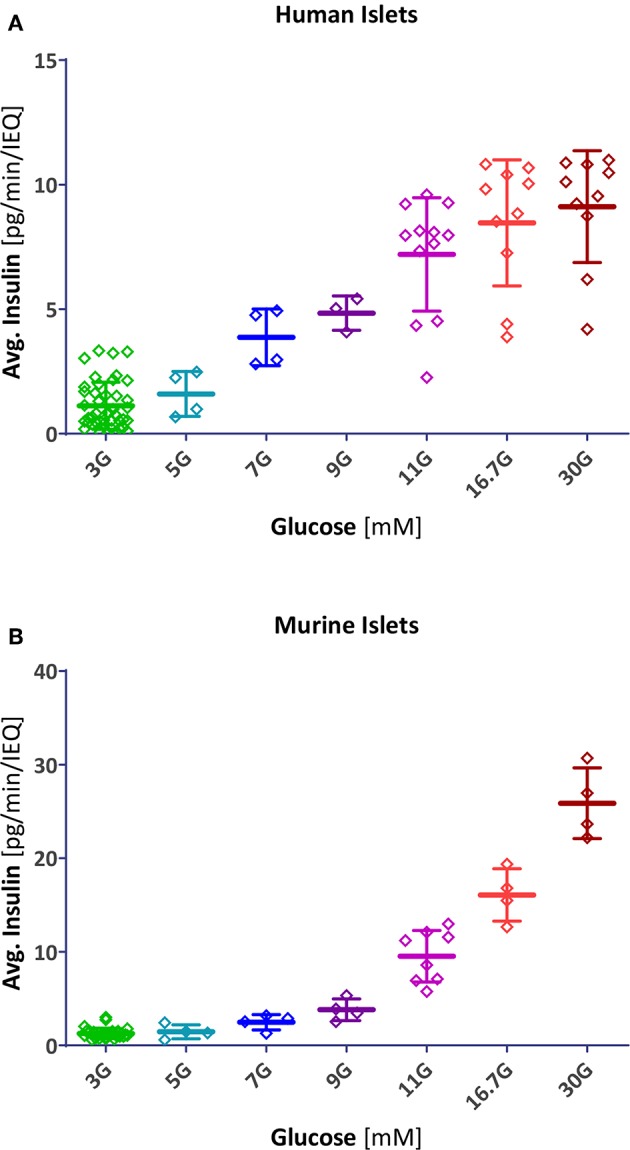
Scatter plot of insulin secretion in response to different glucose concentrations. Data points represent the average insulin secreted by each individual human **(A)** and mouse **(B)** islet sample (pg/min/IEQ; first- and second phase combined). Lines indicate group average and SD.

Concentration-responses for the amount of insulin secreted (Figure 4) could be fitted well with standard sigmoidal functions as represented by the classic Hill function

(1) $$\begin{array}{l} R\left(\%\right)=R/R_{\max}=f_{H}\left(c\right)=\frac{c^{n}}{c^{n}+C_{50}^{n}} \end{array}$$

**Figure 5:**
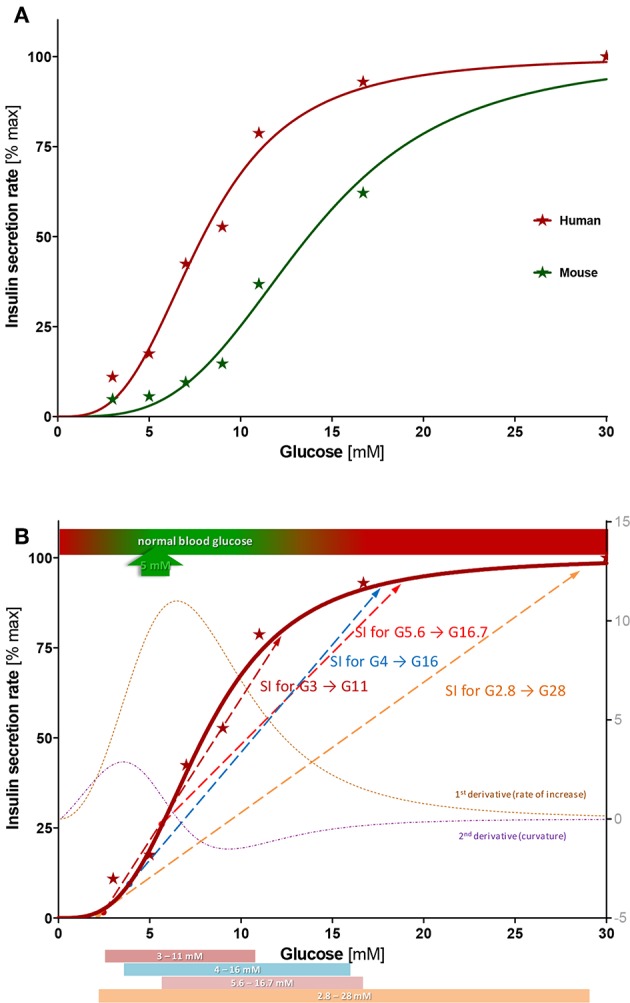
Concentration-response of insulin secretion. **(A)** Average insulin secretion rate (first- and second phase) at different glucose steps expressed as percent of maximum value (at G30) and fitted with a sigmoidal dose response function (Hill function, Equation 1). Compared to human islets, mouse islets have a similar Hill slope (*n* = 3.4 ± 0.4 vs. 3.2 ± 0.4), but a right-shifted response (half-maximal concentration *C*_50_ = 13.7 ± 0.6 vs. 7.9 ± 0.4 mM). **(B)** Best-fit sigmoid function describing percent insulin secretion rate in function of the high glucose challenge for human islets (Equation 1; *n* = 3.2, *C*_50_ = 7.9 mM) overlapped with its first and second derivative (right axis) and some commonly used stimulation indices (SIs). See text for details.

Resulting fits accounted for 98% of the variability in both human and mouse data (*r*^2^ = 0.980 and 0.984, respectively) indicating that this type of function, which has been used for modeling insulin secretion (11, 19), gives a good description (Figure 5A). Compared to human islets, mouse islets have a similar Hill slope (*n* = 3.4 ± 0.4 vs. 3.2 ± 0.4), but a right-shifted response with a half-maximal concentration of *C*_50_ = 13.7 ± 0.6 vs. 7.9 ± 0.3 mM. In general agreement with a previous observation (18), concentration responses of separately calculated first- and second phase releases tracked closely those of the average ones (Figure S3). In these experiments, we obtained maximum insulin secretion rates (for the second-phase response at G30) of 8.3 ± 2.3 and 24.0 ± 5.1 pg/min/islet for human and mouse islets, respectively.

Estimated dynamic stimulation indices (dSI; average values and individual ranges) vs. G3 are shown in Table S1. They were calculated using overall responses, which include both the first-phase peak and the second-phase plateau, but those calculated for second phase responses alone are quite similar (see Figure S3) being only somewhat smaller as they do not include the first-phase peak in their averages. Note that these dSI values are somewhat larger than most previously reported ones because they are sensitive to the value of the denominator (secretion at low glucose), and with the current perifusion machine, we obtained low baseline secretions at G3.

## Discussion

The detailed quantitative characterization of the dynamics of insulin released in response to an increase of glucose concentration is of considerable theoretical and practical interest since this is an essential physiological function necessary to maintain life and the most critical function of pancreatic islets. Here, we collected detailed data using parallel dynamic perifusion studies of isolated human and murine islets under different glucose challenges.

### GSIS Time-Profile: Shape of the Biphasic Response

Insulin secretion in response to a sharp increase in glucose has been long known to be biphasic. This was first suspected based on peripheral and portal blood measurements in humans (20, 21) and confirmed to be so in experiments with perifused rodent pancreas or isolated islets in the late 1960s (5, 7). Such a biphasic response consisting of a transient first phase peak followed by a sustained second phase is now well-characterized in multiple systems (11, 14, 15, 18). While *in vivo* blood glucose levels might never increase fast enough to induce such a biphasic secretion following oral food intake, the pattern is a sensitive indication of adequate β-cell function (22). Whereas, presence of a sharp first phase of insulin release may not be fully evident following an oral meal challenge (as the corresponding increase in plasma glucose is not steep enough), it is clearly present following intravenous glucose tolerance tests (IVGTTs) (23, 24) or in hyperglycemic clamp studies (25) in a manner quite similar to that in perifusion assays. There is evidence that the ability of β-cells to generate a rapidly increasing insulin profile is particularly effective in restraining hepatic glucose production (22), and several studies found an accelerated loss of the first-phase insulin response in those progressing toward T1D (26–29). Hence, presence of an adequate first-phase response is an important consideration in the design of artificial or bioartificial pancreas devices as its lack might have long-term physiological consequences (11, 30).

Here, we found murine islets to respond to a rapid stepwise change in glucose with a much sharper first phase than human ones. They also showed a slowly rising secretion rate during the second phase; whereas, under these *in vitro* conditions, human islets had a completely flat response at all stimulating glucose concentrations. This is in contrast to some *in vivo* findings in humans where hyperglycemic clamps showed increases of plasma insulin values in the second phase (25, 31, 32). However, insulin secretion rates calculated from plasma C-peptide levels by deconvolution had a flat-shaped second phase (32). This discrepancy might be explained, at least in part, by the hepatic extraction and peripheral metabolism of insulin, which may mask the actual secretory rates by the pancreatic islets *in vivo*.

### GSIS Dose-Response (Dependence on Glucose Step-Size)

The nature of the functional form describing the glucose-dependence of the insulin secretion is of considerable interest. Glucose is not a substrate *per se* for insulin production; hence, there is no direct justification for the use of Michaelis-Menten–type enzyme kinetics. Nevertheless, it has been long recognized that sigmoid functions provide good descriptions (14, 15, 33), and a Hill (generalized Michaelis-Menten) equation (Equation 1) provides a mathematically convenient functionality that fits experimental results well. Here, it accounts for 98% of the variability of the data for both human and murine islets (*r*^2^ = 0.980 and 0.984, respectively). A Hill function with *n* > 1 is needed because glucose-insulin response is clearly more abrupt than that of Michaelis-Menten equations (*n* = 1), as illustrated by the sigmoid-type curve obtained here as well as in previous works (14, 15, 34, 35). It has to be noted that Hill functions as used here assume that insulin secretion tends toward zero at low glucose concentrations (Figure 5); whereas, stressed islets tend to leak insulin even at basal glucose (36).

Data here for both human and murine islets (Figure 4) could be fitted very well with Hill type functions having similar slopes (*n* = 3.2 and 3.4); however, human islets reach their half-maximal response much faster than murine ones: *C*_50,gluc_ of 7.9 ± 0.3 vs. 13.7 ± 0.6 mM. It has been suggested that such a shift might be due to differences in glucose transporter expression (37). Interestingly, the responses obtained here agree with recent observations that (transplanted) pancreatic islets are able to set their own glycemic set points, which are ~5 mM for human and ~8 mM for mouse islets (4). These values correspond very nicely with the start of the more abrupt insulin response in our concentration-response curves—being situated in both cases at about 20% of the maximum response and just below the portions with the steepest increase (Figure 5A). Note also that the abruptly rising portion of the response obtained here for human islets overlaps with that of the normal blood glucose concentrations (3–9 mM; Figure 5). For context, we also compared our results (*C*_50_ = 13.7 mM mouse and 7.9 mM human) with data published earlier and, whenever possible, fitted with a Hill function. For rodent islets, earlier works found less right-shifted responses: *C*_50_ = 8.8 mM (*n* = 3.1) for mouse islets (33) and *C*_50_ = 8.2 mM (*n* = 3.6) for perifused rat pancreas (14). More recent mouse data are in much closer agreement with ours: *C*_50_ = 15.6 mM (*n* = 3.7) (38). For human islets, data from the latter group are also in good agreement with ours: threshold at 3–4 mM glucose, *C*_50_ at 6.5 mM, and maximum secretion reached at ~15 mM glucose (Hill-type fit yielding *C*_50_ = 6.7 mM and *n* = 2.3) (35).

### Insulin Secretion Rates

At maximum stimulation (G30), we found human islets to secrete less insulin per unit mass (IEQ) than mouse ones (8.3 vs. 23.9 pg/min/islet) indicating that the maximum secretion rates differ about 3-fold. The amounts of insulin released in response to the KCl-induced depolarization also show a more than 2-fold difference as indicated by the corresponding AUCs: 96.0 vs. 229.2 pg·min/islet. Note, however, that there is considerable variability among samples, and 3–5-fold differences are not uncommon (Figures 2, 4) (9). For example, for the data shown in Figure 2, coefficients of variation (CV% = SD/Mean) for insulin secretion were 48.4 and 43.5% for the first-phase responses at G11 and 38.1 and 55.3% for the second-phase responses of human (*n* = 34) and mouse (*n* = 25) islets, respectively. A larger variability in the second-phase mice data are at least partly due to the rising profile of their response here vs. the flat human one. For the human islets tested here, insulin secreting ability as quantified by the average amount of insulin secreted or the corresponding SI showed no correlation at all with the age (range: 17–66 year) or body-mass index (BMI; range 21.0–36.8 kg/m^2^) of the donor (Figure S4). Note also that while all samples tested here were from donors with no clinical history of diabetes, the presence of recent undiagnosed diabetes cannot be ruled out for some of them (especially since about half had unreported HbA_1c_ levels, Table S3), a possible further confounding factor. Regarding the average insulin secretion rates, the observation of higher insulin secretion in mouse agrees with the overall higher metabolic rates of mice compared to humans and with previous studies suggesting that murine islets contain more β-cells than human ones. For example, mouse islets were found to contain a higher proportion of insulin-containing cells (77 vs. 55%, *p* < 0.05) and a lower proportion of glucagon-containing cells (18 vs. 38%, *p* < 0.05) vs. human (39). Similar proportions (averages of ~85 vs. ~60%) were also found in another study that also indicated declining β-cell proportions in larger human islets (40). Furthermore, while islet insulin content does not seem to be particularly informative regarding GSIS performance neither in human (9) nor in murine islets (41), mouse islets contain more insulin per islet than human ones (18, 38, 42). We did not perform detailed evaluations on these samples, but our results from a smaller set of samples indicate insulin contents of 51.0 ± 9.3 and 10.4 ± 4.1 ng/IEQ for C57BL6 mouse and human islets, respectively.

Overall, the insulin secretion dynamics of human and murine islets were considerably different further supporting the need for detailed studies with human islets (43). Lower insulin release from human islets as compared with murine ones have been observed by others as well. For example, Rorsman and co-workers found secretion values of ~5 vs. ~12.0 pg /min/IEQ at prolonged 10 mM glucose challenge (44), in good agreement with our results here. Dai et al. measured lower first-phase peaks in human islets, ~30 vs. 80 pg/min/IEQ (G16.7) (45). A sharper first-phase peak, an ascending second-phase plateau, and a higher average insulin content in murine islets were also observed by Henquin and co-workers (18, 35, 38).

### GSIS: Stimulation Indices and Assessment of Islet Function

SIs calculated as the ratio between the insulin secretion at high and low glucose are one of the most widely used quantitative descriptors for islet characterization due to their simple nature and independence of islet mass. SIs can be calculated without a need for reference controls and quantification of islet mass. Hence, they are convenient and commonly used. The amount of insulin secreted at high glucose (e.g., AUC) could be a better predictor of islet functionality; however, because it requires islet mass assessment, which is challenging and prone to inaccuracies (12, 13), SIs are more commonly used. Nonetheless, they are sensitive to the value of the denominator (secretion at low glucose), and small changes in baseline secretion, which might be irrelevant to islet function at high glucose, can alter SI values widely. Therefore, SIs can be highly variable from sample to sample as well as from lab to lab; they can range as high as 30 or even more (Table S1) making meaningful comparisons challenging. This is a likely reason why SIs from static GSIS on their own are not good predictors of *in vivo* islet function (46–49).

Furthermore, despite SIs being widely used for islet assessment in both research and clinical applications, there is no uniform standard, and various low- to high-glucose steps are used without clear evidence as to which one provides better characterization of islet function. For example, static GSIS involving a large G2.8 → G28 step is used for clinical (transplantation) assessment (50, 51) as well as for islet assessment by IIDP, but other versions, such as G5.6 → G16.7 (or earlier G5 → G11) (9), G3.3 → G16.7 (49), and several others are also used. For dynamic perifusion our group (DRI, Miami) has been using G3 → G11 (11, 36), whereas G5.6 → G16.7 is used by Powers et al. (9), including the centralized Human Islet Phenotyping Program (HIPP) of IIDP, and different other more or less arbitrary steps are also used.

The present concentration-response study provides information on the suitability of the various glucose-steps used. For human islets, half-maximal response occurs at about 8 mM and the response is essentially saturated by 15 mM. According to the best fit Hill function and its first and second derivatives (corresponding to the rate of increase and the curvature, respectively), the inflection point and steepest increase are around 6.5 mM, $c=c_{50}{\left(\frac{n-1}{n+1}\right)}^{1/n}$, and the strongest upward curvature is at 3.5 mM—just above and below the glycemic set point of ~5 mM (Figure 5B). Overlaying some of the most commonly used glucose steps on this graph highlights some of their main problems (Figure 5B):

- The clinically used G2.8 → G28 step (50 → 500 mg/dL) is too wide, encompassing a large portion of the already plateaued response in normal islets. Hence, it could give an acceptable SI even for islets with a considerably abnormal response with a right-shifted *C*_50_ that start secreting insulin only at abnormally elevated glucose concentrations (e.g., 10 mM). Further, the low-glucose value is too low and close to the secretion-triggering threshold, so that normal islets might secrete very little insulin, while stressed islets might leak insulin making SI highly variable. Hence, variations in basal secretions, which might have no connection to the insulin secretion ability at high glucose, can cause large changes in the calculated SI resulting in uninformative values. In our assays, secretions at G3 ranged from 5 to 25% (vs. maximum at G30) resulting in a large coefficient of variability (CV% = 79%) and dSIs ranging from 4 to 20.
- The smallest G3 → G11 step clearly provides the best fit over the rising portion of the response, where secretion rate increases (Figure 5B); hence, it could provide the most sensitive descriptor. However, it suffers somewhat from the same problem as the previous one: insulin secretion at low glucose is too small and too variable causing inconsistent and possibly uninformatively high SI values. On the other end, its high-glucose step is too low, below where saturation is reached for most islet samples.
- The G5.6 → G16.7 step covers a good range (100 → 300 mg/dL) but misses an important part of the rising segment as it starts at a value that is too high. It is above the average glycemic set point (~5 mM) and at a value (G5.6) where secretion is already around 25% of the maximum (Figure 5B). While this might stabilize the SI fraction, it misses the lower portion of the response where there is a steep increase in secretion rates (Figure 5B).

Based on the data obtained here, we would suggest a dSI with a glucose step covering an increase of (average) insulin secretion from a low of 10–20% to a high of 85–90% as the most reasonable measure to estimate islet function. With the present best-fit Hill function (Equation 1; *n* = 3.2, *C*_50_ = 7.9 mM), this corresponds to glucose concentrations of 4.0–5.1 and 13.9–15.9 mM. Hence, a glucose step of G4 → G16 with 4-fold increase could be a reasonable choice. SI from such a step might be an adequate compromise since it • starts around the threshold of activation and below the glycemic set point, but not from a too low glucose to stabilize SI values, • covers most of the rising, quasi-linear portion of the response for acceptable sensitivity, and • includes part of the saturation-plateau to allow some shift in the *C*_50_ without strongly deteriorating overall SI. By the same logic, a larger high-glucose should be used for assessing the quality of rodent islets that have a right shifted response (larger *C*_50_) compared to human islets; the 10–90% interval would correspond there to 7.2 to 26 mM suggesting, for example, a G7 → G28 step. A brief study suggested G16.5–G19.3 as the high-glucose for SI in rat islets due to its better correlation with viability (52).

As a final note on SI, it should be mentioned that dSI values obtained here (Table S1) are somewhat larger than most previous values reported because with the current perifusion machine, we obtained lower baseline secretions at G3. Most likely, this is because due to the improved design and low perifusion rates of these machines, the islets are experiencing less mechanical stress and are leaking less insulin at low glucose. For example, dSI calculated on the average of all our human perifusions (G3 to G11) is 4.0 (Figure 1) vs. the 7.4 obtained here (Figure 3, G11), mainly because the average low glucose secretion dropped from 1.8 to 1.2 pg/min/IEQ.

## Data Availability Statement

The raw data supporting the conclusions of this manuscript will be made available by the authors, without undue reservation, to any qualified researcher.

## Ethics Statement

The animal study was reviewed and approved by the University of Miami Institutional Animal Care and Use Committee.

## Author Contributions

PB conceived the study, analyzed the data, and wrote the manuscript. OA performed the islet culture and perifusion experiments, analyzed the data, and contributed to the manuscript. Both authors read and approved the final manuscript.

### Conflict of Interest

The authors declare that the research was conducted in the absence of any commercial or financial relationships that could be construed as a potential conflict of interest.
