# Supplementary material for: Concentration-Dependency and Time Profile of Insulin Secretion: Dynamic Perifusion Studies With Human and Murine Islets
Source: Front Endocrinol (Lausanne). 2019 Oct 2;10:680. doi: 10.3389/fendo.2019.00680 (PMC6783504; doi:10.3389/fendo.2019.00680)
Supplement: Supplementary file 1 [file Data_Sheet_1.pdf]

Supplementary Material

Concentration-Dependency and Time Profile of Insulin Secretion: Dynamic Perifusion Studies with Human and Murine Islets

Oscar Alcazar[1] and Peter Buchwald[1,2*]

[1]Diabetes Research Institute, University of Miami, Miller School of Medicine, Miami, FL, USA and [2]Department of Molecular and Cellular Pharmacology, University of Miami, Miller School of Medicine, Miami, FL, USA

1.1 Supplementary Tables

Table S1. Calculated dynamic stimulation indices (dSIs) for the different high-glucose (HG) challenges explored in the present perifusion assay (compared to 3 mM G3 low-glucose baselines) for human and mouse islets. Values are those calculated for the average profiles at each glucose step shown in Figure 3; values below in smaller font indicate range for dSIs calculated for each individual perifused sample.

HG (vs. G3)	Human dSI	Mouse dSI
**G5**	1.6<br>(1.3–5.3)	1.2<br>(1.0–1.7)
**G7**	3.9<br>(2.5–18.8)	2.0<br>(1.1–3.0)
**G9**	4.8<br>(2.7–27.4)	3.1<br>(1.4–8.1)
**G11**	7.1<br>(2.2–30.3)	7.7<br>(1.9–16.2)
**G16.7**	8.4<br>(2.7–29.5)	12.9<br>(11.0–22.9)
**G30**	9.1<br>(2.8–41.5)	20.8<br>(19.7–35.4)

Table S2. Checklist for reporting human islet preparations used in research. Adapted from Hart NJ and Powers AC. *Diabetologia* (2019) 62:212. Data for islets preparations used for the concentration-response dynamic GSIS study (Figure 3A).

**Islet preparation**	**1**	**2**	**3**	**4**	**5**	**6**	**7**	**8**
**Mandatory Information**								
Unique identifier	HP-18196-01	HP-2287	HP-2288	HP-2290	HP-2291	HP-2293	HP-2298	
Donor age (years)	49	63	51	23	45	42	49	
Donor sex (M/F)	M	F	F	M	F	F	M	
Donor BMI (kg/m^2)	30.3	25.1	32.2	21.0	32.1	32.0	25.9	
Donor HbA_{1c} or other measure of blood glucose control	5.8	-	-	-	-	5.8	-	
Origin/source of islets	IIDP	DRI	DRI	DRI	DRI	DRI	DRI	
Islet isolation center	Scharp-Lacy Inst.	DRI	DRI	DRI	DRI	DRI	DRI	
Donor history of diabetes? Please select yes/no from drop down list	No	No	No	No	No	No	No	
If Yes, complete the next two lines if this information is available								
Diabetes duration (years)	-	-	-	-	-	-	-	
Glucose-lowering therapy at time of death	-	-	-	-	-	-	-	
**Recommended Information**								
Donor cause of death	Anoxia	Head Trauma	Stroke	CVA	ICH	CVA	Stroke	
Cold ischemia time (h)	11:09	10:09	14:31	14:10	14:10	10:46	14:38	
Estimated purity (%)	85	90	90	90	80	90	90	
Estimated viability (%)	95	90	90	92	90	90	90	
Additional notes	-	-	-	-	-	-	-	

Table S3. Checklist for reporting human islet preparations used in research. Adapted from Hart NJ and Powers AC. *Diabetologia* (2019) 62:212. Data for islets preparations used for dynamic GSIS study (Figure 2A).

**Islet preparation**	**1**	**2**	**3**	**4**	**5**	**6**	**7**	**8**
**Mandatory Information**								
Unique identifier	IIDP4	IIDP5	HP-2221	HP-2222	HP-2224	HP-2230	HP-2231	HP-2242
Donor age (years)	24	42	23	53	49	52	20	17
Donor sex (M/F)	M	M	F	F	M	F	F	F
Donor BMI (kg/m^2)	29.4	36.8	24.0	34.0	28.1	26.8	30.1	25.6
Donor HbA_{1c} or other measure of blood glucose control	-	-	-	5.3	5.6	6.3	5.4	5.2
Origin/source of islets	IIDP	IIDP	DRI	DRI	DRI	DRI	DRI	DRI
Islet isolation center	DRI	DRI	DRI	DRI	DRI	DRI	DRI	DRI
Donor history of diabetes? Please select yes/no from drop down list	No	No	No	No	No	No	No	No
If Yes, complete the next two lines if this information is available								
Diabetes duration (years)	-	-	-	-	-	-	-	-
Glucose-lowering therapy at time of death	-	-	-	-	-	-	-	-
**Recommended Information**								
Donor cause of death	Head Trauma	Stroke	Stroke	Stroke	CVA	CVA	MVA	MVA
Cold ischemia time (h)	19:37	15:28	21:43	12:47	13:33	12:48	10:14	15:50
Estimated purity (%)	85	95	90	90	80	90	70	80
Estimated viability (%)	93	93	90	93	90	90	90	90
Additional notes	-	-	-	-	-	-	-	-

**Islet preparation**	**9**	**10**	**11**	**12**	**13**	**14**	**15**	**16**
**Mandatory Information**								
Unique identifier	HP-2243	HP-2246	HP-2280	HP-2287	HP-2288	HP-2290	HP-2291	HP-2293
Donor age (years)	39	59	45	63	51	23	45	42
Donor sex (M/F)	F	M	M	F	F	M	F	F
Donor BMI (kg/m^2)	24.8	34.1	24.6	25.1	32.2	21.0	32.1	32.0
Donor HbA_{1c} or other measure of blood glucose control	-	5.7	5.0	-	5.3	-	5.5	5.8
Origin/source of islets	DRI	DRI	DRI	DRI	DRI	DRI	DRI	DRI
Islet isolation center	DRI	DRI	DRI	DRI	DRI	DRI	DRI	DRI
Donor history of diabetes? Please select yes/no from drop down list	No	No	No	No	No	No	No	No
If Yes, complete the next two lines if this information is available								
Diabetes duration (years)	-	-	-	-	-	-	-	-
Glucose-lowering therapy at time of death	-	-	-	-	-	-	-	-
**Recommended Information**								
Donor cause of death	CVA	CVA	Subdural Hygroma	Head Trauma	Stroke	CVA	ICH	CVA
Cold ischemia time (h)	16:25	10:45	12:17	10:09	14:31	14:10	14:10	10:46
Estimated purity (%)	95	90	90	90	90	90	80	90
Estimated viability (%)	93	90	90	90	90	92	90	90
Additional notes	-	-	-	-	-	-	-	-

**Islet preparation**	**17**	**18**	**19**	**20**	**21**	**22**	**23**	**24**
**Mandatory Information**								
Unique identifier	HP-2298	HP-18196-01	10079665	10252228	10374868			
Donor age (years)	49	49	25	55	58			
Donor sex (M/F)	M	M	M	F	M			
Donor BMI (kg/m^2)	25.9	30.3	31.0	35.7	32.4			
Donor HbA_{1c} or other measure of blood glucose control	-	5.8	-	-	-			
Origin/source of islets	DRI	IIDP	IIDP	IIDP	IIDP			
Islet isolation center	DRI	Scharp-Lacy Inst.	Univ. Wisconsin	Univ. Wisconsin	Univ. Wisconsin			
Donor history of diabetes? Please select yes/no from drop down list	No	No	No	No	No			
If Yes, complete the next two lines if this information is available								
Diabetes duration (years)	-	-	-	-	-			
Glucose-lowering therapy at time of death	-	-	-	-	-			
**Recommended Information**								
Donor cause of death	Stroke	Anoxia	Anoxia	CVA	Head trauma			
Cold ischemia time (h)	14:38	11:09	12:30	08:09	06:14			
Estimated purity (%)	90	85	95	94	86			
Estimated viability (%)	90	95	98	98	97			
Additional notes	-	-	-	-	-			

1.2 Supplementary Figures

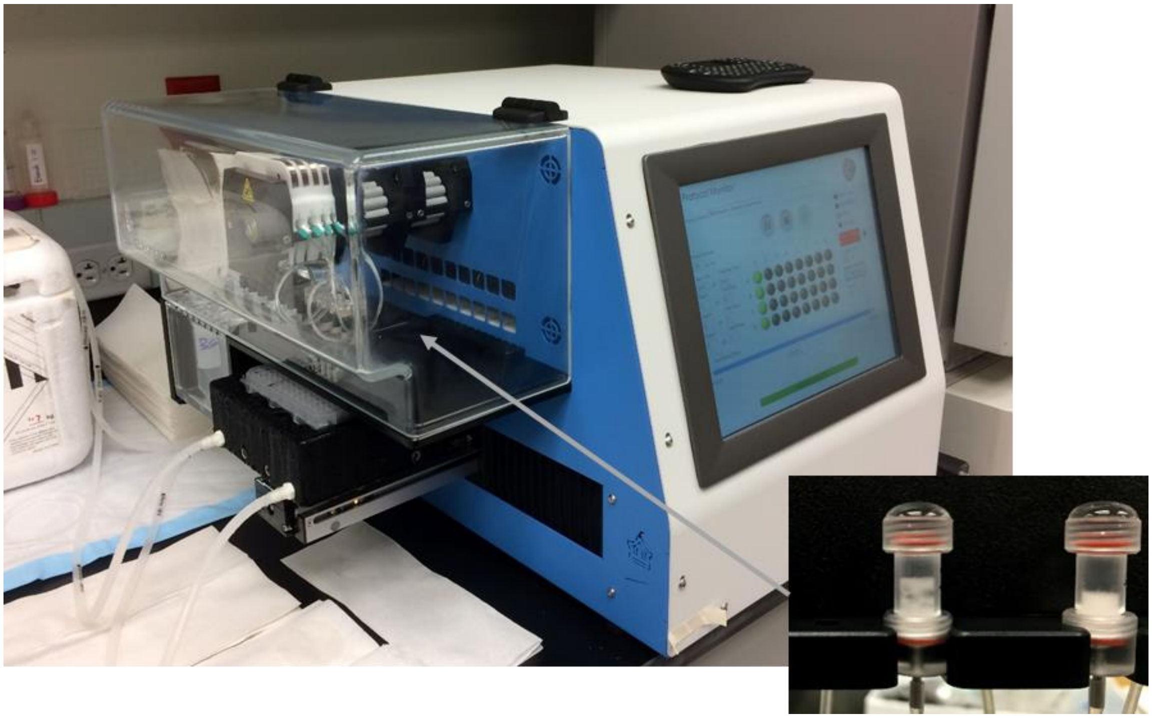

Figure S1. The PERI-4.2 automated perfusion machine (Biorep Inc., Miami, FL, USA) used in the present study with islets in Perspex micro-columns embedded in Bio-Gel P-4 (acrylamide-based) microbead slurry. It allows the dynamic measurement of the glucose-stimulated insulin secretion (GSIS) from isolated islets providing more informative quality assessment than static GSIS/SI. This version has a software-controlled customizable input, multiple (4×3) channels in parallel with adjustable temporal resolution sample collection (e.g., every minute).

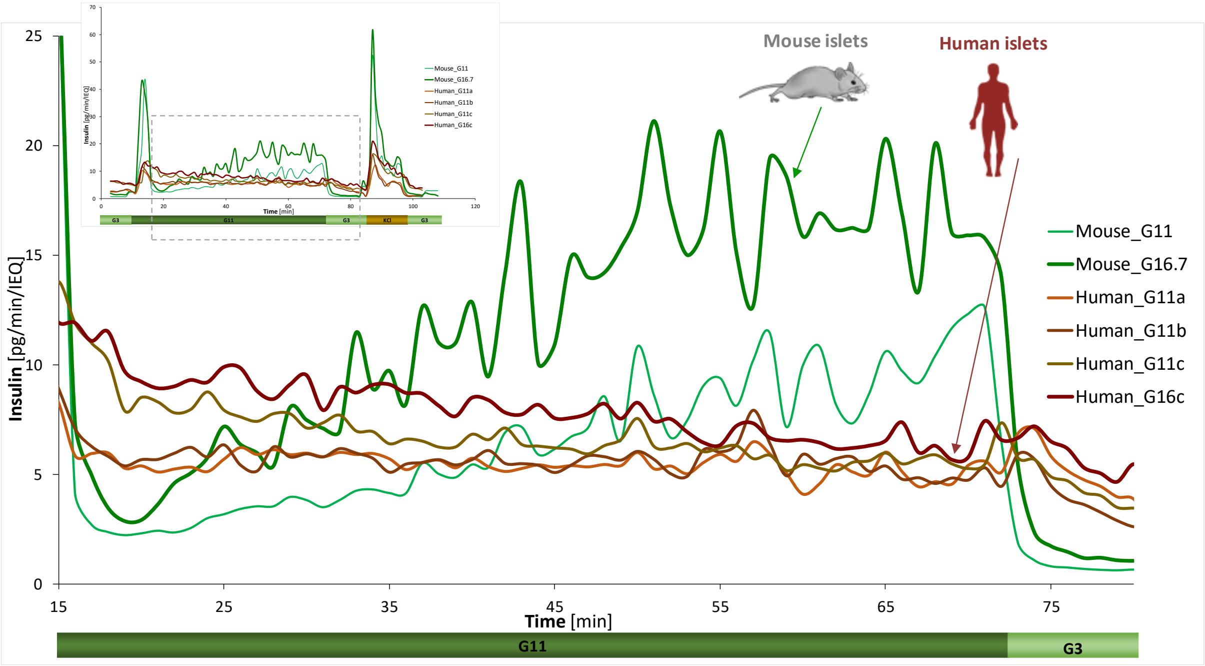

Figure S2. Differences in the second phase insulin secretion of human and murine islets in dynamic GSIS. Data collected for free murine and human islets perifused using a low (G3, 8 min) → high (G11 or G16.7, 60 min) → low (G3, 15 min) incoming glucose stimulation as shown. Data are from single perifusions with prolonged high glucose phase to highlight differences in the second phase of insulin secretion. Insert is secretion profile for the entire perifusion experiment.

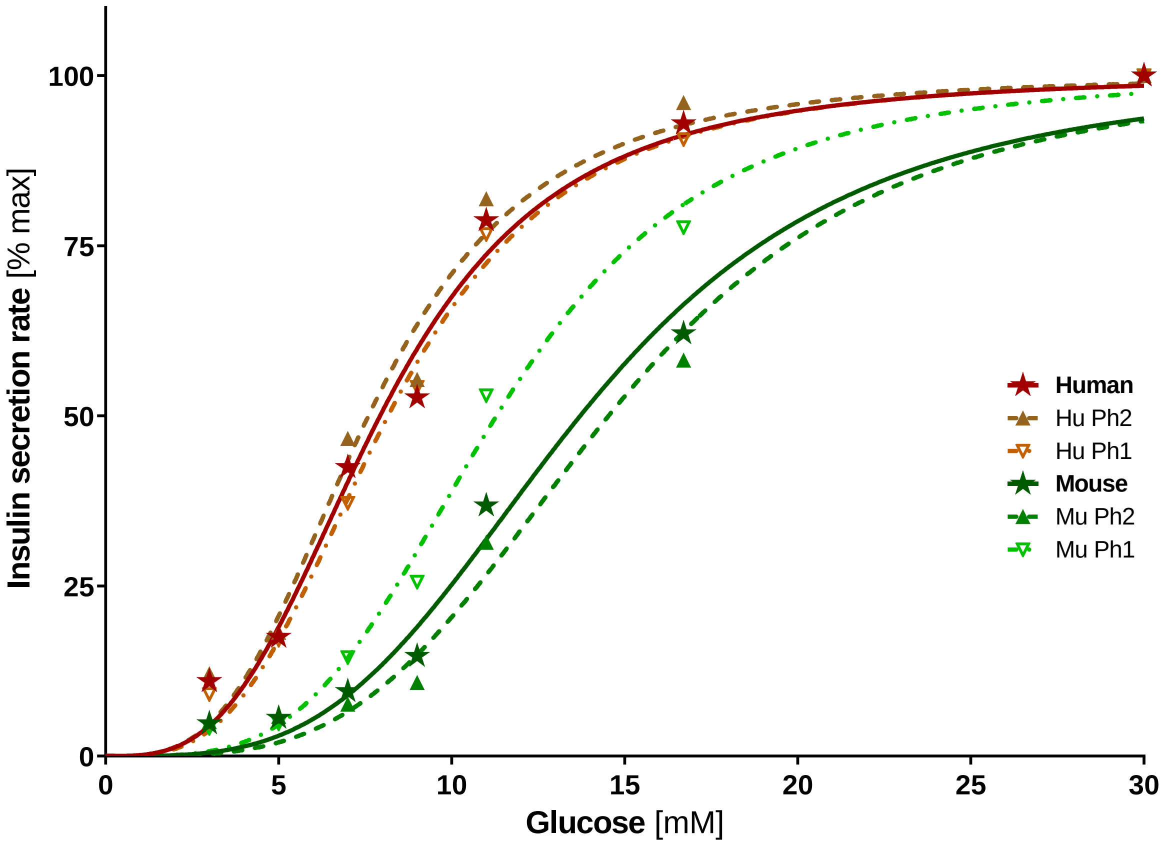

Figure S3. Concentration-response of first and second phase insulin secretion. Average, first-, and second phase insulin secretion rates of mouse and human islets at different glucose steps expressed as percent of maximum value (at G30) and fitted with sigmoidal dose response functions (Hill function, eq. 1).

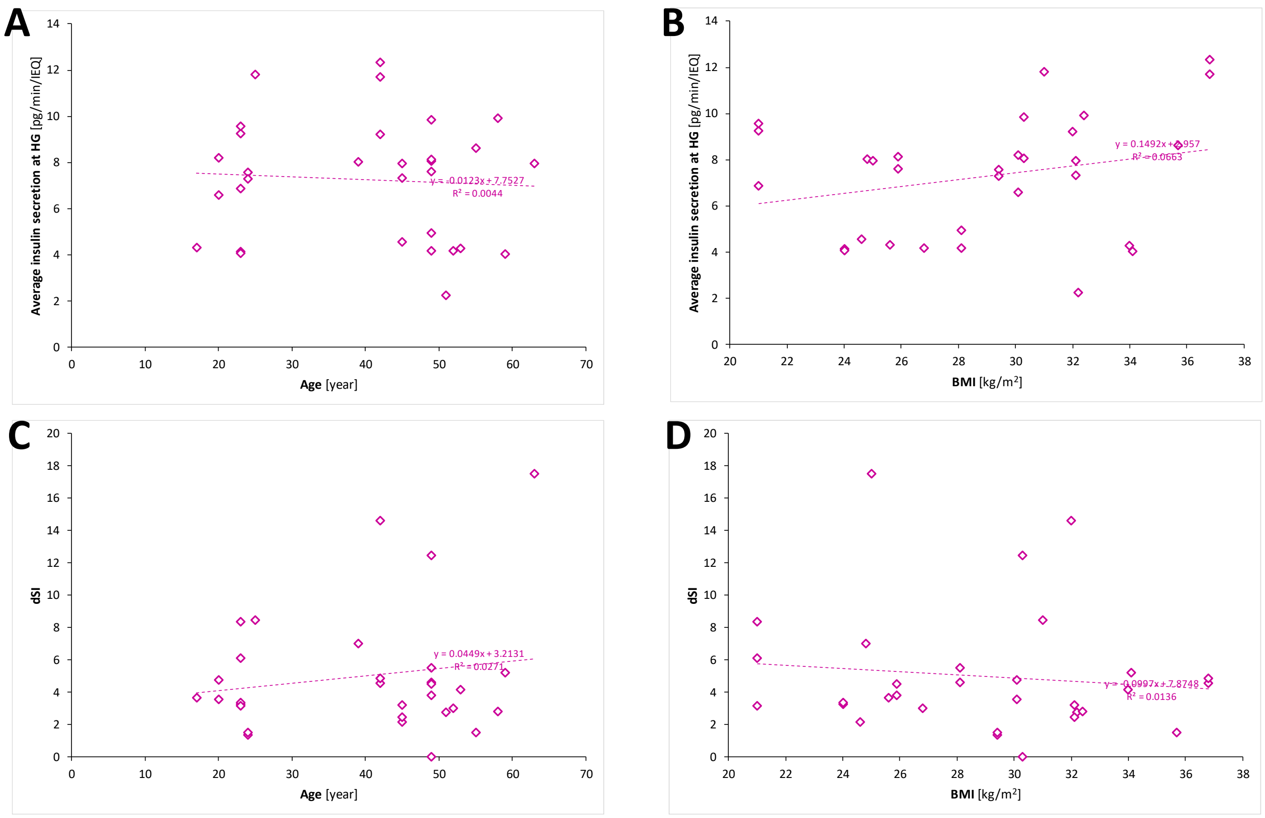

Figure S4. Effect of donor characteristics on insulin secreting ability of human islets. Average amount of insulin secreted at high glucose (G11) and corresponding dynamic stimulation index (dSI) as a function of donor age (**A**, **C**) and BMI (**B**, **D**) for the present dataset (*n*=34). No significant effects were present as indicated by the slope and r^2 values of the corresponding linear regressions.
